# Pulsed Electromagnetic Fields in Bone Healing: Molecular Pathways and Clinical Applications

**DOI:** 10.3390/ijms22147403

**Published:** 2021-07-09

**Authors:** Laura Caliogna, Marta Medetti, Valentina Bina, Alice Maria Brancato, Alberto Castelli, Eugenio Jannelli, Alessandro Ivone, Giulia Gastaldi, Salvatore Annunziata, Mario Mosconi, Gianluigi Pasta

**Affiliations:** 1 Orthopedics and Traumatology Clinic, IRCCS Policlinico San Matteo Foundation, 27100 Pavia, Italy; l.caliogna@smatteo.pv.it (L.C.); medetti.marta@gmail.com (M.M.); alicemaria.brancato01@universitadipavia.it (A.M.B.); albecastell@libero.it (A.C.); eugenio.jannelli@libero.it (E.J.); alessandro.ivone87@gmail.com (A.I.); salvatoreannunziata89@gmail.com (S.A.); mario.mosconi@unipv.it (M.M.); gianluigipasta@yahoo.it (G.P.); 2 Department of Molecular Medicine, University of Pavia, 27100 Pavia, Italy; giulia.gastaldi@unipv.it; 3 Centre for Health Technologies, University of Pavia, 27100 Pavia, Italy

**Keywords:** pulsed electromagnetic fields (PEMFs), biophysical stimulation, osteogenic differentiation, fracture repair, fracture healing, bone regeneration

## Abstract

In this article, we provide an extensive review of the recent literature of the signaling pathways modulated by Pulsed Electromagnetic Fields (PEMFs) and PEMFs clinical application. A review of the literature was performed on two medical electronic databases (PubMed and Embase) from 3 to 5 March 2021. Three authors performed the evaluation of the studies and the data extraction. All studies for this review were selected following these inclusion criteria: studies written in English, studies available in full text and studies published in peer-reviewed journal. Molecular biology, identifying cell membrane receptors and pathways involved in bone healing, and studying PEMFs target of action are giving a solid basis for clinical applications of PEMFs. However, further biology studies and clinical trials with clear and standardized parameters (intensity, frequency, dose, duration, type of coil) are required to clarify the precise dose-response relationship and to understand the real applications in clinical practice of PEMFs.

## 1. Introduction

Pulsed Electromagnetic Fields (PEMFs) are widely used in orthopedic clinical practices to promote bone healing processes [1]. In the 1950s, a group of Japanese researchers discovered the piezoelectric properties of the bone; Fukada and Yasuda demonstrated that in the compression areas the bone is electronegative and causes bone resorption, whereas areas under tension are electropositive and produce bone [2].

Nowadays, bone responses to PEMFs have been widely studied. In the literature, skeletal cells responses to PEMFs have been therapeutically evaluated with devices that expose bone cells to electromagnetic fields in order to stimulate extracellular matrix synthesis for bone and cartilage repair. Understanding the molecular pathways after PEMFs exposure provides important details for their clinical application.

The aim of the review is to highlight the molecular cell responses to PEMFs and their clinical uses in promoting bone repair, tissue engineering and regeneration.

## 2. Materials and Methods

A review of the literature was performed on two medical electronic databases (PubMed https://pubmed.ncbi.nlm.nih.gov (accessed on 3–5 March 2021) and Embase https://www.embase.com (accessed on 3–5 March 2021)) from 3 to 5 March 2021. Three authors performed the evaluation of the studies and the data extraction. All studies for this review were selected by following these inclusion criteria:

- All studies were written in the English language
- All studies were an available full text
- All studies were published in peer-reviewed journals

The study selection and the data extraction were performed independently by three authors. All discrepancies (disagreement) were discussed between the authors and the senior investigators revised the work.

Eligible studies for the review were selected by screening the titles and abstracts using the following three strings, both in PubMed and in Embase:

- bone physiology AND fracture healing.
- electromagnetic field AND fracture healing.
- electromagnetic field AND bone pathway.

In the first string, we selected only the reviews from 2018 to 2020, in the second all the works in the same period and in the third, all works in the last 10 years.

The research using “bone physiology AND fracture healing” produced 155 articles in PubMed and 55 articles in Embase. The two databases share 30 articles. At the end of the reading and screening process a total of 6 articles were identified and selected also by checking the bibliography of all the articles examined.

Using the second strings in PubMed we found 26 articles and in Embase 61 articles. The two databases have in common 23 articles and for this review after reading the texts were selected 2 articles and 1 article were selected checking the bibliography in all articles examined.

The research using the third string produced in PubMed 66 articles and in Embase 65 and the two databases have in common 38 articles. After reading the texts were selected 6 articles and more 10 articles were selected checking the bibliography in all articles examined.

- To evaluate the clinical application of PEMFs the following strings in PubMed and in Embase were used. Fracture healing and magnetic field
- Magnetic field AND delayed union
- Electromagnetic field AND bone healing

All the selected articles of the first two strings were full text, published in the last 10 years (the first two strings), whereas the selected articles from the third string were published in 2019–2020.

The research in PubMed using fracture healing AND magnetic field produced 79 articles and in Embase 91 articles. The two databases have in common 11 articles and for this review after reading the texts, were selected 13 articles and 1 article meeting the inclusion criteria were identified, by checking the bibliography in all articles examined.

The research in PubMed using magnetic field AND delayed union produced 5 articles and in Embase 24 articles. The two databases have in common only one article and for this review after reading the texts was selected only one article identified in the bibliography in the examined articles.

Using the “electromagnetic field AND bone healing” string, we found 32 articles in PubMed and 56 in Embase.

The two databases had 25 articles in common, and for this review two articles were selected.

## 3. Physical Stimulations in Bone Healing

In the last decades, many efforts have been done to understand musculoskeletal tissue regeneration. Biological, chemical, and physiological factors, which play key roles in musculoskeletal tissue development, have been extensively explored. However, the use of physical stimulation is increasing, showing extreme importance in the processes of osteogenic and chondrogenic differentiation, proliferation, and maturation through defined dose parameters including mode, frequency, magnitude, and duration of stimuli.

There are six main categories of physical stimuli involved in bone healing [3]:

1. Mechanical Forces, which direct cellular activities influencing the tissue-level processes of growth, modeling, remodeling, and repair.
2. Ultrasound, that usually refers to a longitudinal wave propagation, a special type of sonic wave with a frequency greater than 20 kHz (this is the upper limit of human audibility), that causes local oscillation of particles. Ultrasound with a frequency around 3–10 MHz is widely used in clinical settings for bone healing.
3. Shock wave, that is a kind of short-duration and acoustic pressure wave consisting of two phases, the positive phase evoking compressive stress (peak pressure: 30–100 MPa) and the negative phase arousing tensile and shear stress (negative pressure). After propagating into tissue, shock waves may lead to microbubble formation of liquid molecules on the focal area, as to increase cell membrane permeability and facilitate the delivery of macromolecules into cells.
4. Scaffold stimulation, which should provide a good environment to guarantee secure attachment, survival, and differentiation of stem cells grown into scaffolds, due to their good osteoconductive and osteogenic ability in bone tissue engineering.
5. Electrical stimulation (EF), which can control and regulate physiologically the cellular and tissue homeostasis. The human body generates a biological EF ranging between 10 and 60 mV at various locations. Furthermore, bioelectricity is very important in the wound healing process. When tissue gets damaged, an EF is created. This endogenous EF causes cell migration to the wound. Indeed, wound healing is compromised when the EF is inhibited. However, the exact mechanism underlying the intracellular signal transduction of Electrical Stimulation in bone repair is still unclear.
6. Electromagnetic stimulation with Pulsed Electromagnetic Fields (PEMFs), focus of this review.

## 4. Pulsed Electromagnetic Fields (PEMFs)

PEMFs are generated from an alternate current being passed through a coil. They are low-frequency magnetic fields with a specific waveform and amplitude, characterized by a constant variation of the magnetic field amplitude over time. PEMFs have been approved by the FDA to treat bone fractures since 1979 as a safe and effective treatment for nonunion of bone, congenital pseudoarthrosis, and failed fusions. Despite its clinical use, cell responses activated by electromagnetic fields in bone tissue are not yet completely known.

Several studies both in vitro and in vivo had been conducted to explore PEMFs effects on osteoprogenitor cells and the skeletal system. The most common cells lines used in vitro are BM-MSCs (Bone Marrow Mesenchymal Stem Cells) and ADSCs (Adipose Derived Stem Cells), while in vivo, the most used models are femoral or tibial osteotomy in rats and rabbits.

Despite numerous studies about the effect of PEMFs stimulations on cells responses, there is no consensus on the optimal parameters (frequency, intensity, and duration) that will promote bone growth and bone healing.

Evidence in literature shows that the most common parameters used both in vitro and in vivo are the following [3]:

- intensity: ranging from 0.1 mT to 2 mT;
- frequency: ranging from 15 Hz to 75 Hz;
- duration: in vitro, the treatment duration ranges from 8 min to 24 h for many days (from 1 to 28 days). In vivo, the treatment duration ranges from 1 h to 8 h for many weeks (from 1 to 12 weeks).

Most in vitro experiments highlighted a gene expression increase of main bone markers alkaline phosphatase (*alp*), runt-related-transcription factor 2 (*runx*-*2*), osteocalcin (*ocn*), and osteopontin (*opn*); then, the enhancement of alkaline phosphatase (ALP)enzymatic activity and other typical bone matrix proteins was also detected.

Moreover, in vivo studies demonstrated that PEMFs have positive effects on bone fractures: a decrease in healing time was observed in different animal models who have had osteotomy.

Due to the central role of Mesenchymal Stem Cells (MSCs) in physiological bone repair, in the last years several studies have been oriented towards the discovering of PEMFs effects on MSCs osteogenic differentiation as well as the signaling pathways involved.

As described below, PEMFs can control the inflammatory microenvironment and promote the MSCs differentiation, playing a pro-osteogenic role.

## 5. PEMFs Molecular Pathways on Bone Healing

The usual bone healing process after bone fracture consists of four distinct phases. However, these stages have considerable overlaps [4]:

1. Fracture and inflammatory phase.
2. Angio-mesenchymal phase.
3. Bone formation.
4. Bone remodeling.

It has been observed that PEMF-activated pathways take a role in bone healing phases 2, 3, and 4, while inhibit the 1 inflammatory phase [5].

### 5.1. Inflammatory Phase and Wnt/β-Catenin Signaling

This stage begins immediately following the fracture (Days 1 to 5). Blood vessels and bone are broken, originating a hematoma around the fracture site. In literature it has been demonstrated an important role and implication of Wnt signaling in the inflammatory phase preceding tissue repair; however, even though the precise molecular network involved is not elucidated yet, in this review we considered Wnt signaling as a key player in the modulation of this early phase. It has been demonstrated that PEMFs were able to activate cell surface adenosine receptor (A2A), resulting in the activation of both canonical (Wnt/β-catenin) and non-canonical (Wnt/Ca^2+^) Wnt pathways [6], as documented by the increased expression of Wnt ligands such as WNT1, WNT3a, and WNT10b in association with increases in both bone mass and strength [7].

Generally, the Wnt ligands activate a series of downstream intracellular signaling pathways: the Wnt/β-catenin, Wnt/Ca^2+^, Wnt/planar cell polarity (Wnt/PCP) or Wnt/protein kinase A (Wnt/PKA) pathways. However, as the canonical Wnt/β-catenin pathway is the most well characterized, for a better comprehension of the molecular mechanisms involved, in this review we will summarize its intracellular cascade [7].

A distinctive feature of the canonical Wnt/β-catenin pathway is the translocation of the β-catenin into the nucleus upon signaling activation. When no Wnt ligands are present, β-catenin is degraded by a β-catenin destruction complex, which includes axin, adenomatosis polyposis coli (APC), protein phosphatase 2A (PP2A), glycogen synthase kinase 3 (GSK3), and casein kinase 1 α (CK1α), whereas the binding of WNTs to the receptor complex, composed by Fz and LRP5/6 co-receptors, triggers a series of events responsible for the degradation of the destruction complex described above. The Wnt binding also cause a diminished axin’ stability in the cytoplasm, and its translocation close to the cell membrane where it interacts with the cytoplasmic tail of LPR5/6 receptor. This event induced the activation of DSH protein which plays a key role in the inhibition of GSK3, protecting β-catenin from its degradation and allowing the protein to accumulate in the cell’s cytoplasm. Then, the stabilized β-catenin enters the nucleus whereby interacting with partner DNA-binding proteins, such as LEF and TFCs, modulate the transcriptional activity of target genes [8].

To conclude, the activation by PEMFs of adenosine receptors, especially A2A and A3, is particularly relevant in this phase as well, since their activation inhibits the NF-kB pathway, a molecular cascade involved in inflammatory processes [1].

### 5.2. Angio-Mesenchymal Phase and VEGF Pathways

In the angio-mesenchymal phase (Days 5 to 11), VEGF regulates the angiogenesis process, which is closely connected to osteogenesis Type H vessels, so named for their high expression of endomucin and CD31, that have recently been identified as able to induce bone formation [9]. The VEGF pathway is the key regulator of vascular regeneration. It has been shown that both osteoblasts and hypertrophic chondrocytes express high levels of VEGF, thereby promoting the invasion of blood vessels and transforming the avascular cartilaginous matrix into a vascularized osseous tissue [10]. VEGF promotes both vasculogenesis, helping aggregation and proliferation of endothelial mesenchymal stem cells into a vascular plexus, and angiogenesis, stimulating the growth of new vessels from the already existing ones. Therefore, VEGF plays a crucial role in the neo-angiogenesis and revascularization at the fracture site. It has been observed that the presence of VEGF promotes fracture healing, while blocking of VEGF-receptors leads to a delay or interruption of the regenerative processes.

Many studies suggested that PEMFs play a promotion effect not only in osteogenesis but also in angiogenesis, in different cellular model both in physiologic and pathologic conditions [11,12]. Therefore, PEMFs may facilitate bone repair by inducing the activation of diverse signaling pathways enhancing both osteogenesis and angiogenesis. Both the FGF and VEGF signaling pathways have been demonstrated to be involved in the regulation of proliferation and differentiation of osteoblasts and in angiogenesis required for bone formation [10]. A study indicated that, in human umbilical vein, after the exposure of endothelial cells (HUVECs) to PEMFs there was a 150% increase of FGF-2 mRNA and a 5-fold rise of the protein, a molecular shift responsible for the augmented endothelial cell proliferation and tubulization, key steps for new vessels formation [13]. The same result has been documented by Delle Monache and colleagues, which revealed that in the same cell type, the PEMFs treatment induced an increase of the protein expression of phosphorylated VEGF receptor 2 (KDR/Flk-1), promoting cell proliferation, migration and tube formation of HUVECs [12].

### 5.3. Bone Formation

In that phase, MSCs previously recruited can differentiate into osteoblasts or chondrocytes to initiate the bone formation. During the process of osteoblast differentiation, RUNX-2 is crucial for the commitment of MSCs to the osteoblast lineage and positively influences early stages of osteoblast differentiation. Osterix (OSX) starts playing an important role in osteoblast differentiation following RUNX2-mediated mesenchymal condensation. During the process of osteoblast differentiation, RUNX-2 induces the expression of bone matrix genes Collagen type 1 (*col1a1*), Osteopontin (*opn*), Bone Sialoprotein (*ibsp*), and Osteocalcin (*ocn*). However, for further bone maturation, *runx-2* expression must be downregulated [14].

At present, the molecular pathways known to be involved in bone formation and activated by PEMFs exposure are as follows:

1. Bone Morphogenetic Protein Signaling Pathway (BMPs) and Tumor Growth Factor β Signaling Pathway (TGF-β)
2. Phosphoinositide 3-Kinases/Akt/mammalian Target of Rapamycin Signaling Pathway (PI3K/Akt/mTOR)
3. Notch Signaling Pathway (NSP)
4. Mitogen-Activated Protein Kinase (MAPK).

#### 5.3.1. TGF-β/BMPs Pathways

TGF-βs and BMPs are cell regulatory proteins belonging to the TGF-β superfamily, which play critical role in the regulation of cell growth, differentiation, and development in a wide range of biological systems. 

Generally, the signaling starts with the ligand-induced oligomerization of receptor kinases and phosphorylation (activation) of the cytoplasmic effectors SMADs (Sma and Mad Related Family): SMAD2 and SMAD3 for the TGF-β pathway, or SMAD1/5/8 for the bone morphogenetic protein (BMP) pathway. Upon phosphorylation, SMADs associate with SMAD4 (co-SMAD), responsible for the whole complex nuclear translocation. Activated SMADs regulate diverse biological effects according to the partner proteins selected. Moreover, the activation of SMADs is balanced by the presence of inhibitory SMADs (SMAD6, SMAD7) whose expression is induced by TGF-β and BMP signaling as part of a negative feedback loop.

##### TGF-β Signaling Pathway

The TGF-β signaling pathway plays an important role in the development, homeostasis, and repair of most tissues in organisms. TGF-β is a potent immune suppressor, and perturbation of the signaling is linked to autoimmunity, inflammation, and cancer [15]. Before binding to its receptors, TGF-β is activated from a large latent complex composed by LTBP (latent TGF-β binding protein) and LAP (Latency-Associated Peptide). Ligand binding to the Type II receptor (TGF-β RII) allows the recruitment and activation of Type I receptor (TGF-β RI). The activated TGF-β RI then phosphorylates its downstream targets SMAD2 and SMAD3 [16] which upon association with SMAD4, translocate into the nucleus, whereby interacting with other transcription factors, regulate gene expression. However, a part the canonical (SMADs-dependent) signaling pathway activation, the TGF-β signaling may trigger SMADs independent pathways too, including Erk, SAPK/JNK, and p38 MAPK signaling.

##### BMPs Signaling Pathway

BMPs (Bone Morphogenetic Proteins) are a large subclass (more than 20 members) of the TGF-β super family acting in many tissues under physiologic conditions. BMPs accomplish their task via receptor-mediated intracellular signaling, ending up with gene expression regulation.

Two types of receptors are required in this process: type I and type II. While there is only one type II BMP receptor (BmprII), there are three type I receptors: Alk2, Alk3, and Alk6. Different combinations of type II and type I receptors determine the outcome of the signaling pathway activation. The canonical BMP pathway acts through receptor I mediated phosphorylation of SMAD1, SMAD5, or SMAD8. Then, two phosphorylated SMADs form a heterotrimeric complex with SMAD4. Finally, the formed heterotrimeric complex translocates into the nucleus and cooperates with other transcription factors to modulate the expression of target genes [17]. Even in this case, a part the canonical pathway activation (SMADs-dependent), the BMPs signals may be transduced thanks to the intricate crosstalk occurring between the BMPs and other important signaling pathways, such as Wnt’s.

Both the TGF-β and BMPs pathways have been found to have a key role in osteogenic process; thus, as a master regulators of bone formation and healing, their activation after PEMFs exposure has been widely investigated. Several studies demonstrated that PEMFs stimulation could significantly increase the expression of TGF-β in osteoblast-like cells [18]. Moreover, another study demonstrated that in human bone marrow stromal cells (hBMSCs), PEMFs exposure was able to activate both the pathways inducing proliferation, differentiation and mineralization of stem cells by up-regulating the gene expression of *runx-2* [19]. The activation of these signaling pathways has been also confirmed by both in vitro and in vivo (clinical trials) studies, as they reported that PEMFs stimulation induced an increased transcription and synthesis of BMPs in an intensity-dependent manner [20]. To conclude, different studies highlighted a synergy between the PEMFs treatments and the administration of BMPs, suggesting that these two stimuli may work on different intracellular pathways, enhancing new bone formation to a greater degree that treating with either stimulus [5].

##### Crosstalk between WNT & BMPs Pathways

Both BMPs and Wnt ligands serve a role in the bone formation, as suggested by a multitude of in vitro and in vivo studies. However, it seems that the crosstalk between these two pathways in bone development is rather complicated, as the two signaling cascades interact differently according to the developmental stage considered [21]. In skeletal development, the mesenchymal precursors undertake the osteogenic differentiation process upon the activation of Wnt/β-catenin pathway; at this stage, the Wnt/β-catenin signaling keep osteoprogenitors dividing preventing their further maturation, a step regulated by BMPs. Therefore, BMP and Wnt signals have opposing effects in osteoprogenitor cells.

However, once osteoprogenitors become osteoblasts, Wnt and BMP signals function cooperatively; both BMP2 and Wnt/β-catenin pathways promote further differentiation, as documented by the expression of ALP and ECM mineralization. However, what is overt is that the outcome of the crosstalk between these two signaling pathways, strongly depends on cell type considered, the step of bone formation or healing, and on the whole cellular and extracellular contexts, as documented by the huge amount of data in literature. This intricate relationship, takes place at different levels, as documented by the regulation of both pathways in extracellular, cytoplasmic, and nuclear contexts.

##### Extracellular Regulation

In the extracellular environment, the binding of secreted molecules to components of both signaling pathways may result either in activation or repression of the two molecular cascades. For instance, the interaction occurring between sclerostin (SOST) with BMPs ligands and/or with the LRP6, prevents the pathways activation. On the other hand, other secreted molecules work by enhancing and encouraging positive interactions between WNTs and BMPs signals [21].

##### Intracellular Regulation

At the intracellular level, the transducer components of both signaling pathways interact with each other’s.

For instance, BMPs inhibit Wnt pathway through a direct interaction between DSH and the phosphorylated SMAD1 creating an inhibitory complex which is broken upon WNT3 stimulation. However, when the cells (bone marrow stromal cells) were treated with both WNT3a and BMP2, the interaction between the two proteins was further enhanced thanks to the phosphorylation of SMAD1.

However, the effect of Wnt signals on BMP pathway is still under deep investigation. To date, there are authors showing an inhibitory effect on BMP signaling [21] and others reporting a synergy between the two [22,23,24,25,26].

Finally, the interaction between β-catenin and inhibitory SMADs might cause either the β-catenin degradation upon ubiquitination or the enhancement of the Wnt/β-catenin signaling pathway’s activation by promoting the formation of β-catenin/LEF-1 transcription complex. Accordingly, the outcome of the relationship depends on the whole cellular context [21].

##### Nuclear Regulation

One of the most compelling evidence of the synergy and crosstalk between these two signaling pathways is the regulation of gene expression at promoter level. As described above, upon signal activation, β-catenin translocates into the nucleus to associate with LEF/TFCs transcription factors, acting as a co-activator. TCFs and LEF contain DNA binding domains able to recognize conserved DNA sequences, a feature shared by the BMP’ signaling effector SMAD4. Indeed, many genes have been found to harbor the DNA binding sites for both the TCFs/LEF complex and SMAD4. In most cases, the gene expression of target genes is synergistically increased, rather than having one stimulus alone. Indeed, when the SMAD4 binding sites were removed from the regulatory region, β-catenin was not able to cause a full transcriptional activity, and the same results were found about the TCFs/LEF binding sites. [21].

#### 5.3.2. PI3K/Akt/mTOR Signaling

The PI3K/Akt/mTOR signaling is a crucial molecular cascade involved in a variety of physiological cellular processes such as cell cycle and metabolism regulation, transcription and translation, cell differentiation, motility, and apoptosis [5]. Indeed, because of its importance in core cell function, its proper signaling controls cell survival.

The mammalian/mechanistic target of rapamycin (mTOR) is a serine/threonine kinase that integrates inputs from nutrients and growth factors to control many fundamental cellular processes through two distinct protein complexes mTORC1 and mTORC2. In particular, mTORC1 has emerged as a common effector mediating the bone anabolic effect of IGF1, WNTs, and BMPs; thus, a dysregulation of mTORC1 could contribute to various skeletal diseases including osteoarthritis and osteoporosis [20]. Indeed, a study published in 2018 revealed that in the presence of an inflammatory environment, after PEMFs exposure the MSC commitment shifted towards an osteoblastic phenotype through the activation of the mTOR signaling pathway [27].

Furthermore, activation of PI3K/Akt signaling in MSCs under PEMFs osteogenic induction has been reported. Zhang and colleagues described increased levels of phosphorylated Akt, phosphorylated GSK3β, and nuclear β-catenin, indicating the Akt/GSK3β/β-catenin axis is involved in osteogenic differentiation, following PEMF exposure [28]. The involvement of Akt has been reported also by Poh and colleagues, who demonstrated that after PEMFs exposure at selected parameters, the activation of Akt in adipose derived mesenchymal stem cells, leads to a significant upregulation of bone-specific genes [29].

#### 5.3.3. Notch Signaling

The Notch Signaling Pathway (NSP) is a highly conserved pathway for cell-cell communication. NSP is involved in the regulation of several processes such as cell differentiation, proliferation, and specification. Actually, NSP is used by a variety of renewing adult tissues to control both the undifferentiated state in the stem cell niche and the cell fate commitment required for tissue homeostasis and renewal (Reactome Notch Signaling). The Notch signaling is activated upon cell-to-cell contact through the interactions occurring between Notch receptors and the ligands Delta and Jagged. The ligand binding induces the cleavage of Notch receptor resulting in the release of Notch intracellular domain (NICD), which translocates to the nucleus acting as a transcriptional co-activator. However, NICD requires a DNA-binding protein, RBP-J (recombination signal sequence-binding protein Jk), to activate the transcription of target genes. In the absence of NICD, the gene expression of its target genes is repressed by RBP-J, thanks to the recruitment of co-repressor complexes, whereas the binding of NICD displaces the co-repressors further allowing the recruitment of co-activator complexes [30].

As a key regulator of cell differentiation, Bagheri and colleagues have found that after PEMFs exposure, the hBMSCs were able to acquire an osteoblastic phenotype through the activation of Notch signaling. Specifically, they reported an increase in the expression of several players acting through the Notch-4 signaling, such as NOTCH-4, DLL4, HEY1, HES1, and HES5 [22]. Moreover, the Notch pathway inhibitors inhibited the expression of osteogenic markers, including DLX5, OSX, as well as HES1 and HES5, indicating that the Notch signaling plays an important regulatory role in PEMFs-induced osteogenic differentiation of hMSCs [5].

#### 5.3.4. ERK/MAPK Signaling

The extracellular signal-regulated kinase 1/2 (ERK) belongs to the mitogen-activated protein kinase (MAPK) family, which plays a role in the signal transduction, conveying extracellular cues towards intracellular targets. These kinases have a variety of intracellular targets, allowing them to control diverse cellular processes, like cell proliferation, cell differentiation, and stress response. The MAPK pathway plays a critical role in PEMFs-induced osteogenic differentiation and osteoblasts’ viability and function. Extremely low-frequency pulsed electromagnetic fields (ELF-PEMFs) treatment could significantly increase the total protein content, mitochondrial and ALP activity, and enhance the formation of mineralized matrix of human osteoblasts with a poor initial osteoblast function, by triggering the ERK1/2 signaling pathway. When the cells were treated with an inhibitor of the ERK1/2 signaling cascade, the positive effects of the ELF-PEMFs treatment on osteoblast function were impaired [31].

Several other studies revealed the involvement of this signaling pathway in the proliferation and osteogenic differentiation of bone marrow derived stem cells, following PEMFs treatment. Specifically, upon PEMFs exposure, the cells displayed an augmented proliferation, an increased expression of some bone specific genes, such *runx-2*, *ibsp*, *opn* and a rise of ALP enzymatic activity [5]. Moreover, also Poh and colleagues have revealed the MAPK/ERK signaling activation after PEMFs exposure. Shortly after PEMFs stimulation it has been detected an increase in phosphorylated ERK1/2, a mechanism involved in cell survival, growth and proliferation [29]. Interestingly, the PEMFs treatment, by activating the ERK and p38 MAPK signaling, was able to modulate both the osteogenic and osteoclastogenic activities necessary for bone homeostasis and physiology [32].

### 5.4. Bone Remodeling

In step 4, the mature osteoblasts begin to produce collagen and calcium deposits (Day 18 onwards, lasting months to years), allowing for the growth of primary bone in fracture site, called woven bone or callus. If the process of union fails, the entire callus becomes fibro-tissue.

A key step of callus remodeling is the establishment of a fine balance between new bone formation, deposited by osteoblasts and bone resorption, executed by osteoclasts.

In physiological conditions, both the processes are tightly regulated to achieve almost-zero changes in bone mass, allowing for bone tissue renewal over time. Both GH and IGF-I play key roles in the regulation of bone growth and homeostasis, thus controlling bone mass. Indeed, GH through direct and indirect (IGF-I mediated) stimulation induce osteoblasts proliferation and activity, promoting new bone deposition; however, its presence also enhances the osteoclasts’ activity and differentiation, causing bone resorption. The result is the increased rate of the overall bone remodeling, with different net outcomes depending on the life stages considered. As a supportive element of the key role exerted by GH, its depletion results in a reduced rate of bone remodeling and a gradual loss of bone mineral density. Specifically, GH directly affects the resident chondrocytes of epiphyseal growth plates, leading them towards the terminal differentiation [33].

#### 5.4.1. GH

GH is a peptide hormone secreted from the pituitary gland under the control of hypothalamus. It exerts direct effect on various tissues including liver, kidney, muscle, central nervous system (CNS), and bone, through the interaction with its membrane receptor (GHR). The GH has two different dependent and independent mechanisms of action, one directly through the GHR and the other inducing IGF-1 secretion. Circulating IGF-1 is mostly synthetized in the liver, but IGF-1 is expressed in all tissues, suggesting that autocrine/paracrine effects of local IGF-1 may be a major mechanism controlling tissue growth. The GHR system utilizes the Janus kinase (JAK) as a signal transducer activating the transcription (STAT) signal transduction pathway [34].

The activated GHR is associated with JAK2, a tyrosine kinase that once activated by GH, phosphorylates STATs-1, -3, -5a, and 5b tyrosine’s. Therefore, STAT proteins translocate to the nucleus where they bind to the specific DNA sequences and activate gene transcription. In addition, recent studies have indicated that suppression of cytokine signaling (SOCS) proteins also controls the GH signaling pathway [34]. These proteins play an important role in growth and skeletal development as well as in inflammation. Chronic inflammation is associated with altered growth and skeletal development, and the SOCS proteins may also have an important role to play in mediating these effects. As GH and IGF-1 have a great effect on bone resorption and bone anabolism, and their administration has a positive effect on osteoporosis and fracture healing, investigating the effects of PEMFs on their effects would be a golden chance to clarify the molecular mechanisms underneath bone healing processes. To date, there are no studies which put in evidence a correlation between the PEMFs exposure and the activation of GH/IGF signaling pathways in the framework of bone repair and healing. Therefore, to take steps forward in the comprehension of PEMFs triggered cellular cascades, it would be of great interest investigating the activation of this pathway too.

#### 5.4.2. IGF

The insulin-like growth factor (IGF) family consists of the ligands IGF-I and IGF-II, the type I and type II IGF cell surface receptors, six specific high-affinity binding proteins (IGFBP-1 to IGFBP-6), IGFBP proteases, and other IGFBP-interacting molecules [14].

The IGF-I is the most abundant growth factor deposited in the bone matrix and stimulates cell proliferation and survival of osteoblasts. The primary function of IGF-1 in the bone matrix is to maintain bone mass and skeletal homeostasis during bone remodeling [35].

Indeed, IGF-1 promotes osteoclast differentiation, through the modulation of RANK and RANKL expression, facilitating the physiological interaction between the osteoblast and the osteoclast [36].

**Figure 1:**
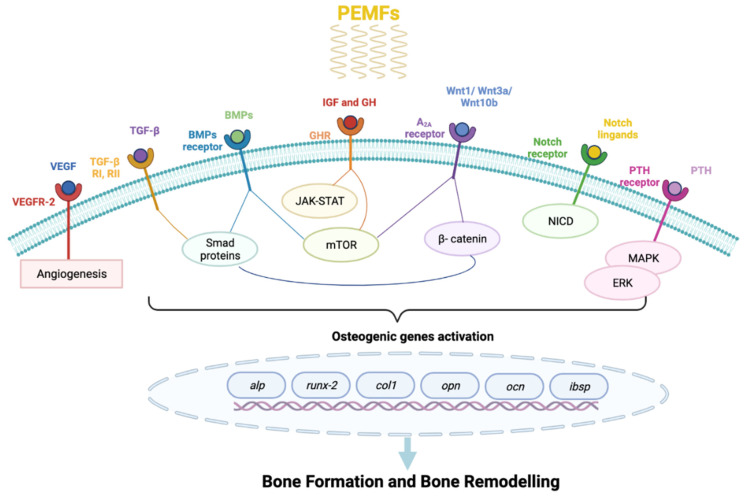
Schematic representation of molecular pathways activated by pulsed electromagnetic fields (PEMFs). Abbreviations: A2A (adenosine receptor); *alp* (alkaline phosphatase gene); BMPs (bone morphogenetic proteins); *ibsp* (bone sialoprotein gene); *col1* (collagen type 1 gene); ERK ( extracellular signal-regulated kinase 1/2); GH (growth hormone); GHR (growth hormone receptor); IGF (insulin-like growth factor); JAK-STAT (Janus kinase- signal transucer activating the transcription); MAPK (mitogen-activated protein kinase); mTOR (mammalian-mechanistic target of rapamycin); NICD (Notch intracellular domain); *ocn* (osteocalcin gene); *opn* (ostepontin gene); PTH (parathyroid hormone); *runx-2* (runt-related-transcription factor 2 gene); SMAD proteins (small mothers against decapentaplegic); TGF-β (transforming growth factor-β); TGF-β RI/RII (TGF-β receptor I/receptor II); VEGF (vascular endothelial growth factor); VEGFR-2 (vascular endothelial growth factor receptor 2).

The results reported above are summarized in Table 1 and Figure 1.

## 6. PEMFs Clinical Effects on Bone Healing

There are tens of thousands of fractures every week in the world, and patients spend billions of dollars a year on treatments [37].

According to the Swedish Patient Register, an estimated 140,000 fractures are treated in Sweden each year. However, national data based on classification and assessments by orthopedic surgeons are scarce [38].

Though bone fracture is a common and costly condition, there is a scarcity of literature focused on the additional costs of healthcare. Cost estimations for fracture healing complications also differ widely in the current literature, depending on the type of complication studied and the method of cost analysis. In UK, a review of evidence on treatments cost for long bone fractures, reported a total cost of £15,566 ($27,100 AUD) for humeral fractures, £17,200 ($29,944 AUD) for femoral fractures and £16,330 ($28,429 AUD) for tibial fractures. In US, treating tibial fractures was estimated as costly as $25,556 USD ($34,472 AUD) per patient, including inpatient, outpatient, and pharmaceutical costs [39].

PEMFs have been widely used to enhance bone repair, accelerating healing process of recent fracture by promoting the callus formation [5], which can be achieved through four distinct phases: inflammatory, angio-mesenchymal, bone formation, and remodeling phases.

There is a lack of consensus in the literature about how to recognize fracture healing, however radiological examinations are often used in the clinical practice. Radiologic evaluation has historically relied upon radiographs and the most commonly fracture healing criteria includes: bridging of the fracture by bone, callus, or trabeculae; bridging of the fracture at three of four cortices; and obliteration of the fracture line and/or cortical continuity [40]. Clinical healing can be defined as the lack of pain and movement at fracture site [41].

A meta-analysis of randomized controlled trials published in 2014 demonstrated a faster healing, expressed in time of radiological union, in patients treated with PEMFs compared with patients treated with placebo, in acute non-operatively treated fractures. However, data from randomized trials were not sufficient to suggest an advantage in using PEMFs in order to reduce the incidence of nonunion in acute fractures [42].

On the other side only three studies out of the sixteen were about PEMFs, hence the authors could not be able to clarify the potential benefits of PEMFs.

Recent data from literature, based on systematic review and meta-analysis of randomized controlled trials, showed an evidence of increased fracture healing rate and reduced associated pain when PEMFs were used; otherwise, there is a lack of evidences regarding the acceleration of the healing time [37].

Hanneman and colleagues in 2012 and 2014 have conducted two randomized controlled trials for non-operative treatment of undisplaced scaphoid fractures [43,44]. The authors suggested that the use of PEMFs in the non-operative treatment of scaphoid fracture had no additional value, but in a well-defined, stable, undisplaced scaphoid fracture, the union can be accelerated.

Adie and colleagues, in a double-blind randomized trial, suggested that PEMFs used as adjuvant of surgery in tibial acute fractures do not prevent secondary surgical interventions for delayed union or nonunion and do not improve radiographic union or patient-reported functional outcomes. However, it should be considered that this study had a short follow-up with low patient compliance (43% of patients provided radiographs at three months, while 36% at six) [45].

Data from literature suggest that the use of PEMFs, as adjuvant in femoral neck fractures fixed with cannulate screws, is able to accelerate fracture healing and to reduce pain [46].

Martinez and colleagues analyzed how electromagnetic therapy can affect the healing of diaphyseal femoral fracture treated with fixation. They showed that PEMFs can promote a faster bone healing [47].

There is a great variability in term of intensity and frequency in each of the reported studies, as shown in Table 2. Actually, further studies are required to analyze and understand the dose-response relationship.

The last phase of bone healing is the bone remodeling phase. In bone remodeling, 5–10% of long bone fracture develop nonunion of fractures [41]. Nonunion occurs when the bone healing process ceases. Identify nonunion in an early stage can be advantageous to limit cost deriving form long period of treatments.

Recently, an Italian group of orthopedic surgeons developed a score, called FRACTING score, which estimates how long the fracture will take to consolidate. The FRACTING score can be employed both to predict months needed for fracture healing and to identify, immediately after operative treatment, patients at risk of prolonged healing. In patients with high score values, new pharmacological and nonpharmacological treatments to enhance osteogenesis could be tested selectively, which may finally result in reduced disability time and health cost savings [48].

PEMFs are an FDA-approved treatment for fracture nonunions [49]. In literature the efficacy of PEMFs in treating tibial delayed unions or nonunions has been reported from 45% to 87% [50].

Cebriàn and colleagues found a rate of union of 91% in patient with tibial pseudoarthrosis, treated by intramedullary nailing and PEMFs, while, in absence of stimulation, the union rate was 83% [51].

A Chinese randomized controlled study investigated the clinical findings of the early application of PEMFs in delayed union of long-bone with a success rate of 77.4% at the end of the study [52].

## 7. Discussion

PEMFs stimulation used for bone repair is widely use in orthopedics clinical practice from nonunion to osteotomy [1].

The early application of PEMFs in fractures that are likely to require a long time to heal is gaining increasing interest [50]. Indeed, the ability to stimulate the healing process locally, without having systemic effects and adverse reactions, is a notable advantage. For these reasons, many efforts have been done in recent years to unravel the molecular mechanisms underlying PEMF-mediated tissue repair and regeneration.

As described previously, our research in the literature highlighted how the PEMFs stimulation always causes the same signaling pathway activation, despite the huge variability between the selected PEMFs physical parameters and the cell lines considered. These results may be indicative of a common conserved response mechanism to physical stimuli. Moreover, even though the bone healing phases display a certain degree of overlapping, the results in literature seem to indicate that the pathways activated upon physical stimulation, were mainly involved in the bone formation phase: in fact, several studies reported that PEMF treatments were able to induce an increase of the TGF-β expression and an augmented proliferation and osteogenic differentiation of stem cells, through the activation of TGF-β, BMP, ERK/MAPK, and Notch signaling [18,19,31,53]. In contrast, the early inflammatory step seems to be attenuated by the influence of PEMFs stimuli. The inflammatory response triggered by the rupture of blood vessels and bone, plays a crucial role in bone healing allowing for the recruitment of cells necessary for tissue repair and regeneration. In the literature, it is reported that the application of PEMFs may help in modulating the inflammatory response, due to both the activation of Wnt and the inhibition of NF-kβ signaling upon ARs stimulation and the activation of mTOR signaling, responsible for the regulation of cellular differentiation [1,27,54]. However, as the molecular pathways strongly interact with each other’s, a deeper spatial-temporal analysis of the pathway’s activation and inhibition would help in the comprehension of the complex cellular responses. Finally, in our work we depicted the role of GH and IGF-I in bone growth and remodeling, required for the mature bone homeostasis. However, in the literature, data about the role of PEMFs on this phase are missing; therefore, for this reason, its investigation should be considered.

To sum up, all the data reported in literature give a solid base for the clinical application of PEMFs; unfortunately, the selected electromagnetic field parameters are very different (frequency, waveform, and amplitude), thus preventing the possibility to carry out accurate analysis. Despite this variability, the intense efforts done were able to decipher, at least in part, how PEMFs could interact with the cellular physiology. However, limitations regard the scarce pool of molecular pathways investigated. Indeed, to deepen the knowledge about the cellular responses to PEMFs stimulation, broader investigations are required. For instance, it would be interesting analyzing in vivo the cell survival, apoptosis, epigenetic changes, and stress responses as a way to have a broader look into the whole cellular context and on pathophysiology of the tissues. Thus, a better comprehension of the in vitro effects of PEMFs on biological systems would be a golden chance to foresee the in vivo outcomes, where the pathophysiological dynamics are much more complex. Unfortunately, to date, there is a great heterogeneity of the PEMFs physical parameters used, both for in vitro and in vivo studies. As a consequence of lack of standardized experimental guidelines, controlled trials resulted with non-comparable and inconclusive data.

## 8. Conclusions

Further in vitro studies and clinical trials with clear and standardized parameters (intensity, frequency, dose, duration, and type of coil) are required. Indeed, it is necessary to clarify the real dose-response relationship to understand the plausible PEMFs applications in the clinical practice, while also allowing a better management of financial resources in healthcare systems.

## Figures and Tables

**Table 1:** Molecular pathways activated by PEMFs exposure.

Pathway Activated	Cell Response	Bone Healing Phase
β-Catenin/Wnt	NF-kβ inhibition *col1* and *opn* increase	phase 1- inflammatory phase; phase 4- bone remodeling
FGF and VEGF	endothelial cells and osteoblastic stimulation	phase 2- angio-mesenchymal phase
TGF-β/BMPs	*runx-2* increase	phase 3- bone formation
PI3K/Akt/mTOR	osteoblastic genes activation	phase 3- bone formation
Notch	osteoblastic genes activation	phase 3- bone formation
ERK/MAPK	osteoblastic genes activation	phase 3- bone formation
GH/IGF	JAK-STAT activation	phase 4- bone remodeling

**Table 2:** Each single study had their parameter in terms of frequency, dose, and duration.

Study	Field of Application	PEMFs (Device)	Frequency, Dose, Duration
Adie et al.	Adjuvant in surgery (tibial shaft)	EBI Bone Healing System (Biomet, New Jersey)	10 h/day 12 weeks
Faldini et al.	Adjuvant in surgery (femoral neck fractures)	Biostim (Igea, Carpi)	75 Hz, 2 mT 8 h/day 90 days
Hanneman et al. (2012)	Acute scaphoid fractures	Ossatec (Uden)	24 h/day 6/12 weeks
Hanneman et al. (2014)	Acute scaphoid fractures	Ossatec (Uden)	24 h/day 6 weeks
Martinez-Rondanelli et al.	Adjuvant in surgery (Diaphiseal femoral fractures)	Authors provided	5–105 Hz, 0.5–2.0 mT 1 h/day 8 weeks

## Abbreviations

A2A	adenosine receptor
A3	adenosine receptor
ADSCs	adipose-derived stem cells
ALP/*alp*	alkaline phosphatase protein/gene
Alk2	activin receptor-like kinase-2
Alk3	activin receptor-like kinase-3
Alk6	activin receptor-like kinase-6
APC	adenomatous polyposis coli
BM-MSCs	bone marrow mesenchymal stem cells
BMPs	bone morphogenetic proteins
BMP RI	bone morphogenetic protein receptor I
BPS	bone sialoprotein
CD31	cluster of differentiation 31
CK1	casein kinase 1
c-fms	colony-stimulating factor-1 receptor
CNS	central nervous system
COL1/*col1*	collagen type 1 protein/gene
DSH	disheveled
EF	electrical stimulation
ELF-PEMF	extremely low-frequency pulsed electromagnetic field
ERK	extracellular signal-regulated kinase 1/2
FGF	fibroblast growth factor
FGF-2	fibroblast growth factor 2
Fz	frizzled
GH	growth hormone
GHR	growth hormone receptor
GSK3	glycogen synthase kinase 3
hBMSCs	human bone marrow stromal cells
HUVECs	human umbilical vein endothelial cells
IGF	insulin-like growth factor
IGFBP	Insulin-like growth factor binding protein
JAK	janus kinase
KDR/Flk-1	phosphorylated vegf receptor 2
LAP	latency-associated propeptide
LEF	lymphoid enhancer factor family
LIPUS	low-intensity pulsed ultrasound
LRP	low-density lipoprotein receptor-related protein
LTBP	latent TGF-β binding protein
MAPK	mitogen-activated protein kinase
MCSF	monocyte/macrophage colony-stimulating factor
MSCs	mesenchymal stem cells
mTOR	mammalian/mechanistic target of rapamycin
NF-kB	nuclear factor kappa-light-chain-enhancer of activated B cells
NICD	notch intracellular domain
NSP	notch signaling pathway
OCN/*ocn*	osteocalcin protein/gene
OPN/*opn*	osteopontin protein/gene
OSX/*osx*	osterix protein/gene
PCP	planar cell polarity
PEMFs	pulsed electromagnetic fields
PI3K/Akt/mTOR	phosphoinositide 3-kinases/akt/mammalian target of rapamycin
PKA	protein kinase A
PP2A	protein phosphatase 2 A
PTH	parathyroid hormone
RANK	receptor activator of nuclear factor κ B
RANK-L	nuclear factor kappa B ligand
RUNX-2/*runx-2*	runt-related transcription factor 2 protein/gene
SAPK/JNK	stress-activated protein kinase/c-Jun NH_2_-terminal kinase
SMAD	small mothers against decapentaplegic
SOCS	suppression of cytokine signaling
SOST	sclerostin
STAT	signal transducer activating the transcription
TCF	T cell factor
TGF-β	transforming growth factor-β
TGF-β R I	transforming growth factor-β receptor I
TGF-β R II	transforming growth factor-β receptor II
VEGF	vascular endothelial growth factor
VEGFR-2	vascular endothelial growth factor receptor 2
